# Using a Robot to Address the Well-Being, Social Isolation, and Loneliness of Care Home Residents via Video Calls: Qualitative Feasibility Study

**DOI:** 10.2196/59764

**Published:** 2025-05-08

**Authors:** Lise Birgitte Holteng Austbø, Ingelin Testad, Martha Therese Gjestsen

**Affiliations:** 1 Centre for Age-Related Medicine, Stavanger University Hospital, Stavanger, Norway; 2 Department of Clinical Medicine, University of Bergen, Postboks 7804, Bergen, 5020, Norway, 0047 40063238; 3 University of Exeter Medical School, Exeter, United Kingdom

**Keywords:** care home, geriatric, aging, dementia, user experience, social isolation, loneliness, feasibility, robot, video calls, mobile phone

## Abstract

**Background:**

About 40,000 people are living in Norwegian care homes, where a majority are living with a dementia diagnosis. Social isolation and loneliness are common issues affecting care home residents’ quality of life. Due to visitation restrictions during the pandemic, residents and family members started using digital solutions to keep in contact. There is no framework or guidelines to inform the uptake and use of technologies in the care home context, and this often results in non-adoption and a lack of use after the introduction phase. Hence, there is a great need for research on the feasibility of a robot that can facilitate video communication between residents and family members.

**Objective:**

This study aimed to (1) introduce video communication through a robot to address social isolation and loneliness in a care home during a period of 6 weeks and (2) identify elements central to the feasibility concerning testing and evaluating the use of the robot.

**Methods:**

Three focus group interviews were undertaken: 1 with family members (n=4) and 2 with care staff (n=2 each). The informants were purposely selected to ensure that they had the proper amount of experience with the robot to have the ability to inform this study’s objectives. The focus group interviews were tape-recorded and transcribed verbatim, then subsequently analyzed using systematic text condensation.

**Results:**

The data analysis of focus group interviews and individual interviews resulted in three categories: (1) organizing the facilitation of video calls, (2) using a robot in dementia care, and (3) user experience with the robot.

**Conclusions:**

Video communication in care homes is a feasible alternative to face-to-face interactions, but it depends on organizational factors such as information flow, resources, and scheduling. In dementia care, the user-friendly robot supports person-centered care through tailored social interaction. Both family members and staff express enthusiasm for video calls as an option and see its potential for future use.

## Introduction

### Background

About 40,000 people are living in Norwegian care homes [1], and about 84% of them are living with dementia [2]. Most residents also have medical comorbidity along with complex care needs due to a combination of cognitive, functional, and communication impairments and neuropsychiatric symptoms [3]. Social isolation and loneliness are common problems [4-6], and these aspects were exacerbated because of the infection control measures (eg, visitor restrictions) undertaken in response to the COVID-19 pandemic [7,8]. As a consequence of the social distancing, families started using digital solutions to keep in touch with their loved ones residing in care homes [9-11]. In the present literature, there is a large number of small-scale studies that explore the use of technologies for care home residents, their families, and staff. For example, digital communication solutions (eg, tablets with video and sound; telepresence robots) for interactions between patients and family [12,13]. There is a general notion that the “digital revolution” in health care also includes long-term care, but there is no framework or are no guidelines to inform the uptake and use of technologies in the care home context [14-16]. Hence, there is a need for systematic research on the feasibility of such solutions in terms of factors affecting the uptake and use, and also to inform future research in this field [17].

### Informing a Digital Intervention in Care Homes

Despite the rhetoric associated with the benefits of digital solutions in health care, the uptake and use have not progressed at the pace and scale anticipated [18]. Limited fidelity of technology recommendation to a person’s needs may be one reason, but it is increasingly recognized in the research literature that the health care personnel’s acceptance of the technological application itself remains a key challenge in adopting an intervention [19,20]. This underlines the vital importance that the involved stakeholders (eg, researchers, policy makers, health care personnel, patients, and carers) can judge the value of technologies in their own right. Conversely, until we develop solutions that are considered to be useful and fit for purpose by the actual users, we will repeat what has been observed, analyzed, and conceptualized by Greenhalgh and Abimbola [21], in their Nonadoption, Abandonment, Difficulties in Scaling Up, Spread, and Sustainability framework: problems in technology projects usually occur because they are too complex and because the complexity is suboptimally handled.

Independently from the COVID-19 pandemic, but capitalizing on the experience accumulated, the application of digital solutions in a care home context has the potential to improve the quality of care as well as the quality of life for each resident. A recent review by Knapp et al [22], provides an overview of digital solutions that appear most ready for use in the next 5 years: digital care for tailored strategies for carers; mobile technologies for supporting self-care and daily activities; touchscreen and multimedia interventions and activities to improve mood, engagement, and behaviors; and ICT-based technologies for social connection.

During the periods of COVID-19 lockdown in care homes in Norway, staff described the need for a solution for residents and their family and friends to keep in contact while visits to the care homes were restricted. Through a collaboration with a cluster of technology vendors (Norwegian Smart Care Cluster), we identified a secure digital solution that could be tested for this purpose.

The digital solution entails using a robot to address social isolation and loneliness. The robot is developed in collaboration with care home staff and residents, and family members, and consists of an iPad mounted on a wheeled stand or a tripod suitable for tabletop placement. The technology enables video communication similar to platforms such as FaceTime (Apple Inc) or Skype (Microsoft Corp), allowing for 2-way communication between residents and family members who have the family member application compatible with the robot downloaded on their smartphone. Family members can maneuver the robot from their phone; however, this feature was not used in this study. The robot is not equipped to interact with residents through voice prompts. The technology is integrated and developed on a secure health platform developed and provided by PatientSky (EG Norge) to ensure patient privacy.

The study applies the New Medical Research Council guidance for developing and evaluating complex interventions [23] in the process of developing an intervention entailing the use of a robot in care homes. The New Medical Research Council’s framework is recommended for the development of interventions containing several interacting components, which aligns well with the multitude of stakeholders and number of components involved when a digital solution is tested in primary care. This study reported in this paper pertains to the first steps in the framework, which are development and piloting. Development phases and feasibility or pilot studies are recommended before conducting larger evaluation studies of complex interventions, to explore the procedures and applicability as well as participants’ experiences with the intervention [24,25]. This can facilitate improvements and inform the design of potential future confirmatory studies.

### Study Aims

This study aimed to (1) introduce video communication through a robot to address social isolation and loneliness in a care home during 6 weeks and (2) identify elements central to the feasibility in terms of testing and evaluating the use, that is, how recruitment and inclusion can be optimized, which outcome measures related to the intervention are feasible, and how care staff and family members experienced using the robot as a video communication tool.

Experiences and findings related to the first 2 elements (ie, how recruitment and inclusion can be optimized, which outcome measures related to the intervention are feasible) are applied when we are currently planning a randomized controlled trial using the same intervention. This paper reports on the care staff and family member perspectives and experiences with using the robot in video communication. The findings can help inform other researchers who potentially face similar challenges when developing digital interventions in a similar setting.

## Methods

### Context

This study is a result of the collaboration between researchers or authors, and members in a care home research network called Fokus, geographically located in Western Norway. The Fokus network supports research activity within care homes, with the overall aim of improving the lives of people in care homes, with particular focus on individuals with dementia. Designed to be mutually beneficial, the Fokus network aims to enhance the quantity and quality of practice-adjacent research within care home settings, increase the accessibility of research to primary care, but also vice versa: primary care stakeholders are vital to researchers per developing knowledge that is relevant in their day-to-day practice. Fokus currently consists of 20 care homes in the southern part of the Western Norway Regional Health Authority. As part of this collaborative network, this feasibility study was conducted in 2 units in one of the Fokus care homes; a dementia care unit and a short-term care unit.

### Intervention

The robot was introduced to the care staff and patients, and was used for 6 weeks. Care staff and family members were introduced to the robot and received training before starting the project. Patients and family members communicated through the robot regularly, but the robot did not replace actual visits. It is estimated that the robot was used 1‐2 times a week per patient, and the conversations lasted for approximately 15‐30 minutes and were facilitated by care staff to ensure no technological difficulties. The entire staff at the 2 units was encouraged to interact with the robot as much as possible to gain experience.

### Study Design

This is a 6-week feasibility study to test and describe the use of a robot for video calls in a care home setting. This study will help inform how recruitment and inclusion can be optimized and assess the feasibility and practicality of measuring the outcomes of the intervention in this context. We used trusted, validated questionnaires from the residents for this purpose, however this article will focus on the care staff and family member perspective and experience with using the robot in video communication. Data was collected using focus group interviews [26].

### Recruitment

As this is a feasibility study, no formal sample size calculation was required. We purposely recruited 2 care home units, 1 dementia care unit, and 1 short term care unit in a care home from the Fokus network. Care staff were recruited from the network members, who further identified residents and family members from their respective units and determined their eligibility to use the robot. Recruitment was conducted in May and June of 2020. Inclusion and exclusion criteria was designed to be as inclusive as possible, and only to exclude individuals who would not be able to use the intervention: (1) all individuals residing in participating care homes who have severe dementia corresponding to the score of “3” or greater on the Clinical Dementia Rating scale, (2) not able to undertake activities in daily living, and (3) any resident from whom consent or the advice of a consultee could not be obtained. A total of 5 residents used the robot for video communication with their respective family members. Four staff members were designated users and facilitators of the communication.

### Data Collection

Three focus group interviews were undertaken: 1 with family members (n=4) and 2 with care staff (n=2 each), respectively. The informants were purposely selected by the head of the care home units, to ensure that they had the proper amount of experience with the robot to have the ability to inform this study’s objectives. Although 5 residents used the robot, 4 family members participated in the interviews, as 1 family member had a conflicting schedule at the time of the interview. The interviews were conducted by a moderator (LBHA) and comoderator (MTG). The administrative coordinator of the Fokus network participated in one of the staff interviews. All interviews were based on a semistructured interview guide focusing on the staff’s and family members’ direct experience with the robot, as well as their take on promoting and hindering factors to the implementation and use of such a measure in a care home setting. The interview with family members and the first staff interview were conducted through Zoom and the second interview with staff was conducted in the care home; all 3 were audio recorded. All interviews lasted for about 60‐70 minutes.

### Analysis

**Figure 1.**
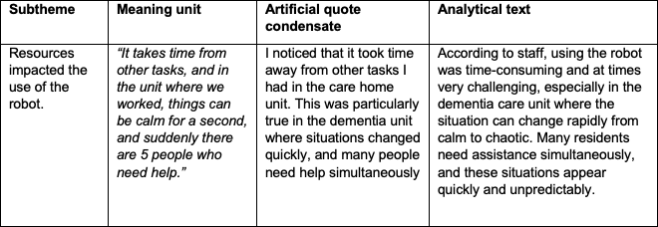
Analytical process.

The focus group interviews were transcribed verbatim immediately after (by LBHA) and analyzed by all authors according to systematic text condensation as described by Malterud [26]. This method includes 4 steps to convey the participants’ experience with a phenomenon. First, all authors read the transcripts to form an overall impression and identify main themes. Second, meaning units were derived from the transcripts and coded into subgroups based on the themes from the first step. Third, the meaning units were written into artificial quotes called condensates. As the last step of the analysis, the condensates were written into analytical text and represented as results. The analytical process is demonstrated in Figure 1.

### Data Saturation

Data saturation was discussed during analysis and was considered obtained when no more information could be attained and further coding of the material was not feasible.

### Ethical Considerations

This study was reviewed by and obtained ethical approval from the Regional Ethics Committee South East D (#15405). Informants provided a written consent and were informed that they could withdraw from this study at any point and without reason. Qualitative data from the interviews were transcribed verbatim and anonymized by replacing informants’ names with a number. All data were collected and stored per data protection regulations; they were stored electronically on computers, which were access-controlled via passwords. Hard copies of transcripts were securely stored in locked filing cabinets in offices that were accessible only to research staff. Data were deleted at the end of this study. The participants were not compensated for their participation.

## Results

### Cohort Characteristics

All care staff and family members who had experience using the robot were invited to focus group interviews, and 8 participants consented to participate. Care staff and family members were interviewed separately; 2 staff members from a dementia care unit participated in the first interview, 2 staff members from a short-term care unit participated in the second interview, and 4 family members participated in the third interview. The interviews were conducted in the fall of 2020. Care staff included nurses, and nursing and medical doctor students. The family members were children and spouses of the residents who had been using the robot. There were no exclusions or losses of participants after consent to participate in the interviews.

The data analysis of focus group interviews resulted in three categories: (1) organizing the facilitation of video calls, (2) using a robot in dementia care, and (3) user experience with the robot. Content from step (4) in the analysis (recontextualization) is presented as analytical text with category headings, respectively, and assembled with quotes that are representative of the category.

### Organizing the Facilitation of Video Calls

#### Overview

Findings from the focus group interviews showed that organizational factors affected the use of the robot for video calls. Adequate information was paramount to involve staff, resource barriers impacted the use of the robot, and structuring the video calls to accommodate routines was essential to the facilitation. Subthemes within this theme are related to information, resources, and scheduling.

#### Care Staff Information Needs and Superusers

Care staff highlighted the need for extensive information and orientation before implementing the robot in their daily routine and care. Findings demonstrate that a well-planned introduction focusing on organizational leadership and motivated staff is crucial to successful use.

*It is very important that everyone who will be involved and use the robot is involved from the very beginning and receives information*.

[Staff from the dementia care unit, focus group 1]

At first, all staff were motivated and engaged with the project. However, after the introduction phase, 2 superusers in each unit were responsible for using the robot and facilitating video communication. This resulted in a lack of enthusiasm from the rest of the staff. Care home leaders must drive the implementation and use of digital solutions to ensure optimal uptake.

#### Resources Impacting the Use of the Robot

According to staff, using the robot was time-consuming and at times very challenging, especially in the dementia care unit, where the situation can change rapidly from calm to chaotic. Many residents need assistance simultaneously, and these situations appear quickly and unpredictably.

If you do not have the time or resources […], it highly depends on your work shift. During the daytime, I never have the time.

[Staff from the dementia care unit, focus group 1]

Staff explained that assisting residents using the robot took time away from other responsibilities. Yet, they acknowledged that the robot would be a great relief if residents could use it independently. They also highlighted that clarifying the care home unit’s capacity concerning time and technology knowledge is key before implementing such a solution.

#### Scheduled Use of the Robot

Staff concurred during the interviews that facilitating video communication with residents’ family members without a scheduled time was time-consuming. Establishing regular communication with family members was emphasized as key to using the robot beneficially.

Many people have one-to-one care, and staff members always have to be present, so it is more about making everything work with the daily tasks.

[Staff from the dementia care unit, focus group 1]

Making video call appointments allowed staff to plan and prioritize resources to have enough capacity to support the resident during the calls.

Communication with family members and scheduling calls. It was time consuming when the participants could not use the robot by themselves.

[Staff from the short-term care unit, focus group 2]

[Staff from short-term care unit, focus group 2]

This was also a great help in ensuring the robot was used regularly. Both family members and staff expressed that they, as care partners, need to be involved in planning to ensure that using a robot is not seen as a burden.

### Using a Robot in Dementia Care

#### Overview

Introducing a robot intended to support person-centered care and social interaction for people with dementia requires thorough evaluation and assessment. This will uncover the benefits and consequences of using the robot, and help understand how to fully use it. In this main theme, the subthemes are people living with dementia using the robot and a user-friendly robot in dementia care.

#### People Living With Dementia Using the Robot

Informants reveal that using the robot for people with a dementia diagnosis pinpoints new opportunities and challenges. Care staff observed that their residents enjoyed seeing their family members through video and that the experience brought instant pleasure and joy.

I could see that the resident was smiling, the instant joy was most definitely present, and he did not become agitated afterwards.

[Staff from the dementia care unit, focus group 1]

In addition to enjoying the actual video conversation with their family members, the robot seemed appealing to residents with a previous interest in computers. Despite having advanced dementia and a reduced understanding of the concept of conversation, some residents were still fascinated with the technology.

Although care staff saw benefits for their residents, they stressed the importance of evaluating cognitive and functional status before using the robot for residents with dementia. This was to identify residents who may benefit from using it. One resident became agitated and uneasy after using the robot to talk to their family, thus requiring follow-up from the staff, who expressed the importance of a well-planned routine when ending a video conversation.

#### User-Friendly Robot in Dementia Care

Care staff found the robot easy to use and very user-friendly. Being part of a project using a digital solution was a good experience, and they said that the robot was a great tool in dementia care. However, implementing technology brought some skepticism, especially regarding technology replacing human contact and in-person visits.

You do not want to replace visits with a screen because it is not the same. However, I did not feel that that changed.

[Staff from the short-term care unit, focus group 2]

In the first weeks of this study, the care home used the robot on a stand with wheels. However, they experienced some challenges using this in a dementia unit and switched to using the robot on a stand for tabletops. There were minor issues with the internet while trying to connect to family members, yet this did not affect the usage.

### User Experiences With the Robot

#### Overview

Family members were enthusiastic about using the robot and saw it as a good solution for communication during the pandemic. Staff expressed a positive attitude after using the robot for 6 weeks and highlighted essential aspects for practical implementation and use. The subthemes under this main theme are family member experience and care staff experience with video calls, respectively.

#### Family Member Experience With Video Calls

There was a general agreement that the family members appreciated the chance to see their loved one. Hearing their voices and seeing their facial expressions gave family members a safe feeling and made it easier for them to get a sense of the residents’ state of health.

Because phone calls are just a conversation, but when you can see them as well, you feel more of a connection.

[Family member, focus group 3]

For my mom, I think this went well, and I thought it was great to see her, and she could see us. Other family members came onto the video, saw her and waved at her. We found much joy in it.

[Family member, focus group 3]

They also pinpointed the robot’s usefulness in communicating with distant relatives, allowing the whole family to gather.

#### Care Staff Experience With Facilitating Video Calls

Introducing the robot to care home staff created both skepticism and enthusiasm. Staff were motivated and excited to try new technology and saw it as a possible solution for relief on a busy day. However, some staff experienced a mixed reception and acceptance among their colleagues. As some residents became agitated and uneasy after using the robot, staff lacked the motivation to use it and saw it as a burden. On the contrary, some did not see it as a burden, but it could be demanding to familiarize themselves with the robot and incorporate it into their daily tasks:

Using a solution like this is something that cannot be forced. There has to be a need for it.

[Staff from the short-term care unit, focus group 2]

A well-planned introduction and adequate information during start-up are key. Sufficient information in the beginning and frequent follow-up during the 6 weeks is crucial to staff feeling motivated to use the robot. The robot will only be used to its full potential if they have a sense of ownership of it.

Information and follow up of the care staff is very important when using the robot [...] if not, it is easy to think “Well, this does not entail me. I have enough to deal with, with my own tasks and patients.

[Staff from the dementia care unit, focus group 1]

During the pandemic, the informants pointed out that they saw a clear need for a solution like the robot in this study. Care home staff had to assume a role as a link between residents and family members. They had to care for residents unable to receive visits from their families and simultaneously take care of family members who did not have the opportunity to follow up with their loved ones.

We had to take on the role of a family member and caregiver simultaneously, as well make sure to update the residents’ family [...] It became very busy for us [...] it would be great to have this opportunity, and great if everyone had one (a robot) in their room.

[Staff from short-term care unit, focus group 1]

There was a general agreement that technology acceptance might be influenced by generation. Some are worried about using digital solutions to deliver care and the distance between caregiver and resident that might arise:

They are anxious to lose the well-established human contact in care.

[Staff from the short-term care unit, focus group 1]

They suggested that future care home staff may have different perspectives on the integration of technology into their work.

## Discussion

### Principal Findings

In this study, using a robot for video communication between care home residents and family members was feasible and provided an alternative to face-to-face communication during visitation restrictions. However, the feasibility was substantially influenced by organizational factors such as information flow, available resources and scheduling video calls with family members. Using a robot in dementia care can support person-centered care by facilitating social interactions, as long as the use is adapted to individual needs. The robot was user-friendly, and both family members and staff expressed enthusiasm to have video calls as an option. This paper provides new insight into using a robot in a care home context, based on the user perspective. This study bridges an existing gap in the literature and has the potential to inform future research aiming to measure the effectiveness of technology on quality of life and loneliness among care home residents.

In the care home context, the introduction of the robot elicited information-related needs from the care staff to maintain encouragement and motivation to use the robot. Novel technology in health care services is often associated with a lack of use after the introduction phase [27,28]. This became evident in this study as facilitation tasks were predominantly obtained by a few individuals in each care home unit after the start-up. Ko et al [14] states that designated superusers appear to be in a better position to support technology implementation, compared to the rest of the staff. However, little is known about the selection of these superusers to optimize uptake. In our study, the designated staff were preselected and received training in facilitating video calls through the robot to involve the care home unit. They, however, seemed to carry out the task mostly by themselves. An issue that might be solved with a continuing flow of information to keep the remaining staff motivated.

Even though a positive attitude toward the robot was evident, available resources and capacity to fully engage and incorporate the solution into everyday care emerged as an obstacle. Despite the understanding that lack of use is often due to unmet expectations or lack of clear value to the user [18], external promoting and hindering factors can contribute. This was evident in our study as the staff clearly stated that the capacity to implement a robot needs to be evaluated at the organizational meso level to ensure optimal use.

Facilitating video communication in daily life unveiled both opportunities and challenges, especially finding the time to call family members alongside daily routines. Staff started to engage family members and schedule calls to accommodate resource challenges. The agreement between family members and staff about involvement in setting up calls enforced collaboration and was seen as a benefit. Revealing the engagement on both sides, we can argue that the robot brought forward an enthusiasm, regardless of skepticism and attitudes toward new technology. Unlike our findings, previous research reports that scheduling calls was a major source of frustration, as this made the opportunity for communication more rigid [9]. This further emphasized the need for family involvement to ensure that using a robot is not seen as a burden.

The introduction of a robot for care home residents with dementia displayed both benefits and disadvantages for the residents. It necessitates a comprehensive evaluation that takes into account the unique needs of each resident to complement person-centered care. Aligned with other research, staff reported that some residents had adverse reactions after the video call and became uneasy and confused [11]. A study [11] found that the appearance of the device caused confusion and anxiety, however, this stopped once the conversation started, and the resident could see their family. Additionally, a sudden disconnect in the technology, where the conversation ended abruptly left the resident anxious and upset. Similar to this, our results showed that the agitation started when the conversation came to an end. It seems that the transitional phase between the start and end of a video call, and the appearance and disappearance of family members on the screen, is grounds for distress. This makes it evident that new technology has to be fit for purpose and its intended users [21,29].

The robot’s user-friendliness resulted in positive experiences when facilitating video calls. Yet, some were still skeptical and concerned that the technology would replace human care. These are aspects that are highly important to take forward when planning implementation and use of technology in health care systems, as these are features highlighted as attributes affecting adoption and future use of new technology [29].

Having video calls as an alternative during visitation restrictions revealed a notable level of enthusiasm among family members and a positive attitude among care staff regarding the robot’s role in facilitating communication during the pandemic. These findings underscore the importance of investigating both user perspectives to comprehensively assess the feasibility and effectiveness of such technology in health care settings.

While dealing with the reality of not being able to visit residents in care homes, family members expressed gratitude toward the given opportunity to maintain social interactions through the robot. Restrictions and lack of social interaction were a major issue in care homes during the pandemic, and video calls were care home residents’ main source of social enrichment during the pandemic [9]. The ability to hear their voices and observe their facial expressions through the use of technology engendered a profound sense of safety among family members. Kelly et al [9] found that adding a visual aspect to the communication made a substantial difference to their experience. Moreover, this gave family members a better understanding of the resident’s overall state of health.

Various emotions and reactions arose from the staff; some were enthusiastic, and some expressed skepticism toward the robot. The enthusiasm was grounded in excitement about integrating technology into their existing routine and saw it as an addition to high-quality care. This is consistent with a finding from a review by Ko et al [14]; care homes are using technology as supplements and not replacements for “human care.”

New technology needs to have clear value for staff, but the most important evident value is for the residents [27,28]. As a consequence of some residents becoming agitated after a video call, certain staff members were discouraged from using the robot and perceived it as a potential burden. This observation underscores the importance of individualizing solutions in the field of care to prevent any adverse effects on the user, and for staff to observe the value of the robot in its own right [18].

Care home staff found themselves assuming a dual role of caregivers for residents who could not receive visits from their families, and facilitators of communication between residents and their loved ones. Most residents in Norwegian care homes have a dementia diagnosis, which is already associated with burnout and depression in care home staff, as caregiving for this group is demanding [30]. This added workload intensified the demand for such technological tools. Notably the informants in this study were interviewed while the Norwegian government still had imposed guidelines and restrictions in terms of social distancing for individuals with increased risk. This is important in the consideration of this paper and the generalizability of the results.

### Strengths and Limitations

This study contributes novel information about the feasibility of a robot in a care home setting through the exploration of the user perspective. The results will emphasize an unmet knowledge need in the field of technology in dementia care, as well as inform future research and guide the implementation of similar digital solutions. Although the total number of informants was small (n=9), the group of participants varied with a wide range of working experience and experience from being a family member to a person residing in a care home, making the group representative of stakeholders of a care home.

The studies reliability was maintained through an accurate and well-described performance of interviews and the analysis. All researchers sought to approach the data with an open mind to investigate the phenomenon purely from the perspective of the participants. Our methodological approach was suited to the aim of the study, to explore the feasibility of the robot based on the user perspective, ensuring the validity of our results. Interviews with care staff and family members were based on similar interview guides, only to differ in wording to fit their role toward the patients, and were performed by the same researchers to ensure internal validity. Preliminary results were distributed and presented to the informants for discussion and validation.

### Implications

This study provides novel information and insight about the use of a robot for video communication in a care home context, as described by care staff and family members. Evidence based on technology implementation in health care mostly concerns non-adoption, abandonment, and poor uptake, making our results paramount in future research and implementation planning. One of the main challenges with existing implementation processes is the “top-down” perspective. Implementation strategies are commonly described by policymakers and researchers, but rarely in collaboration with the users. Stakeholders are key partners in technology implementation, and their acceptance and perceptions are of utmost importance to successful use.

Our results suggest that using video communication in a care home context has the potential to impact loneliness, social isolation by providing a communication alternative when face-to-face visits are not an option. According to previous research, being able to maintain social interaction with family increases quality of life [31]. By studying and describing the feasibility of using a robot for video communication, we can ensure innovative and more efficient pathways for planning and implementing technology in a care home context without disturbing well-established routines, ultimately benefiting patient care and staff satisfaction.

This paper emphasizes the importance of further research, both investigating the effect of using a robot in a care home context, focusing on the reduction of social isolation and loneliness and further describe factors promoting and hindering implementation.

This paper highlights the complexity of introducing a robot in care home context. It underscores the critical roles of leadership, resource assessment, structured scheduling, and person-centered evaluation in ensuring a successful and meaningful integration. As the landscape of care continues to evolve, understanding what factors influence the feasibility of implementation and use of a robot in care homes will be pivotal in enhancing the quality of care provided to individuals living with dementia. Further research and ongoing adaptation will be necessary to address the evolving needs and expectations of care home residents and their families.

### Conclusion

This study suggests that using a robot for video communication in care homes is a feasible alternative to face-to-face interactions during visitation restrictions. Feasibility depends on organizational factors such as information flow, resources, and scheduling. In dementia care, the user-friendly robot supports person-centered care through tailored social interaction. Both family members and staff express enthusiasm for video calls as an option.
